# Risk factors for severe hand foot mouth disease in Singapore: a case control study

**DOI:** 10.1186/s12879-015-1195-2

**Published:** 2015-10-31

**Authors:** So-Phia Chew, Shu-Ling Chong, Sylvaine Barbier, Aji Matthew, Jan Hau Lee, Yoke Hwee Chan

**Affiliations:** Department of Emergency Medicine, KK Women’s and Children’s Hospital, 100 Bukit Timah Road, Singapore, 229899 Singapore; Centre for Quantitative Medicine, DUKE-NUS Graduate Medical School, 8 College Road, Singapore, 169857 Singapore; Department of Children’s Intensive Care, KK Women’s and Children’s Hospital, 100 Bukit Timah Road, Singapore, 229899 Singapore

**Keywords:** Hand foot mouth disease, Enterovirus-A71, Risk factors, Encephalitis

## Abstract

**Background:**

Hand foot mouth disease (HFMD) is a common childhood infection that can potentially lead to serious complications. The aim of this study is to identify risk factors of acquiring severe HFMD in our population.

**Methods:**

We performed a case control study using patients admitted to our hospital from August 2004 to July 2014. Cases were patients with severe HFMD disease while controls were age-matched patients obtained from the same year, in a 2:1 ratio. Data comprising demographic characteristics, clinical symptoms and signs, and lab findings were collected. Conditional univariable logistic regression was performed to determine risk factors for severe disease.

**Results:**

A total of 24 cases of severe HFMD were identified and matched with 48 controls. Seventeen (70.8 %) cases had central nervous system complications. Seven (29.2 %) had cardiovascular complications without evidence of myocarditis. One patient died of encephalitis. The overall mortality of severe disease is 4 %. Evidence of hypoperfusion, seizure, altered mentation, meningeal irritation, tachycardia, tachypnea, raised absolute neutrophil count and EV-A71 (Enterovirus A71) positivity were significantly associated with a severe course of HFMD.

**Conclusion:**

In managing children with HFMD, physicians should consider these factors to help identify patients at risk for severe disease.

## Background

Hand foot and mouth disease (HFMD) is a common childhood infection characterized typically by fever with mouth ulcers, eruption of vesiculo-papular rash over the hands, soles and/or buttocks. It is caused by a group of enteroviruses, most frequently Coxsackie A16 and Enterovirus-A71 (EV-A71). The mode of transmission of HFMD is mainly via the fecal-oral route, respiratory droplets, contact with blister fluid of an infected individual or general close contact with infected individuals. HFMD is a significant public health disease with epidemics reported frequently throughout Asia [1].

While the majority of HFMD cases are mild and self-limiting, severe complications such as encephalitis, meningitis, acute flaccid paralysis, myocarditis and pulmonary edema have been reported [2–4]. These complications may result in significant morbidity or even mortality. In recent years, outbreaks of HFMD have been increasingly reported in eastern and southeastern Asia, including Singapore [5], Malaysia [3], Vietnam [6], mainland China [7] and Taiwan [4]. The clinical challenge for physicians caring for children with HFMD is the difficulty in predicting which child will develop a severe course of disease. This is due to the non-specific complaints of children and their variable clinical presentations. Previous studies, largely from China, have been performed to identify risk factors associated with severe HFMD [5, 7–11]. The only local study was done more than a decade ago [5].

Over the last decade, nationwide epidemics in Singapore were observed in years 2002, 2005, 2006 and 2007 [1]. Despite stringent measures taken in pre-school centres to prevent the transmission of infection in our country, the annual incidence rate of HFMD per 100,000 population had increased from 125.5 in 2001 to 435.9 in 2007 [12].

In this study, we aim to identify risk factors of acquiring severe HFMD in our population. These would be categorized firstly as information readily available from history and physical examination, and then other laboratory predictors when available and accessible. We hypothesized that lethargy, vomiting, raised neutrophil count and EV-A71 infection are risk factors for severe HFMD.

## Methods

This age-matched case–control study was approved by the Singapore Centralised Institutional Review Board (CIRB) E (Paediatric Medicine). We obtained cases and controls from a retrospective chart review of patients admitted with the International Classification of Diseases (ICD) code for HFMD from August 2004 to July 2014. HFMD was clinically defined as the presence of papular/vesicular skin rash on the hand, feet/buttocks and oral ulcers in association with an acute prodrome of fever [1]. A case subject was defined as a child with severe disease characterized by any of the following clinical outcomes: death, encephalitis, meningitis, myocarditis, pulmonary edema or acute flaccid paralysis (AFP). Encephalitis and meningitis were characterized by abnormal neuroimaging or postmortem examination, biochemical abnormalities on cerebrospinal fluid, clinical documentation of meningeal irritation, altered mentation or seizures. Myocarditis was characterized by raised cardiac enzymes or abnormal echocardiography. We included patients with hemodynamic instability who required intervention consisting of fluid resuscitation or inotropic support. Pulmonary edema was defined by the presence of pink frothy secretions on intubation or changes on the chest X-ray (CXR). AFP was characterized by the acute onset of areflexic limb weakness. Controls were obtained from patients admitted with the ICD code of HFMD, in the absence of severe disease. Controls were matched in the ratio of 2:1 to cases for the year of presentation and age.

Data comprising demographic characteristics, clinical symptoms and signs, and laboratory findings were collected through review of medical records. The following symptoms were included: duration of fever, maximum temperature recorded, presence of vomiting, oral ulcers, rashes on the palms and soles, seizure, headache, history of altered mentation, reduced feeding and evidence of hypoperfusion as characterized by lethargy, syncope or reported cool extremities. Vital signs were recorded and compared to age-dependent normal ranges. The following clinical signs were collected: presenting (and any deterioration of) Glasgow Coma Score (GCS), presence of seizure activity on arrival, signs of altered mental status, poor peripheral perfusion, meningeal irritation, paralysis and distribution of rashes. Laboratory findings studied included: total white cell count with absolute neutrophil and lymphocyte counts and EV-A71 status of stool, throat swab and cerebrospinal fluid samples.

We summarized continuous variables in medians and inter-quartile ranges (IQR). We reported categorical variables as absolute numbers and proportions. Categorical variables were analyzed by Fishers’ Exact test and continuous variables were analyzed by the Mann Whitney *U* test. We performed a conditional univariable logistic regression to find predictors that were discriminatory for severe disease. Statistical significance was established as *p* < 0.05 and the data were analyzed using STATA v12 (Stata Corp, College Station, Tx, USA).

## Results

We identified a total of 24 cases of severe HFMD disease during the study period, which were matched with 48 controls. The median age of our patients (*n* = 72) was 2.4 (IQR 1.3-5.4) years. Among the cases, the median length of hospital stay was 4 (IQR 3–7) days. Six children (25.0 %) were admitted to intensive care unit (ICU) and the length of stay ranged from 1 to 10 days. The patient with only 1 day of ICU stay died shortly after admission. Out of those who were admitted to ICU, 5 children required intubation for prolonged seizure or poor GCS. Thirteen (54.2 %) patients required fluid boluses at the emergency department (ED) in view of tachycardia, hypotension or signs of poor peripheral perfusion. Among those who received fluid boluses, the median average fluid volume received was 20 (IQR 10-40 ml/kg and two of them required inotropes subsequently.

Seventeen (70.8 %) cases of severe HFMD were complicated by central nervous system (CNS) disease, with encephalitis being the most common complication. Among those with CNS complications, 6 patients suffered residual neurological deficit on discharge. Two of them developed cerebellar signs while another 2 had lower limb weakness with speech issues. One patient suffered cortical blindness with motor deficit and 1 had right lower limb weakness. Two patients with CNS complications had cardiopulmonary collapse. One was successfully resuscitated. The other patient who died had an accelerated course of illness. He presented with severe dehydration on day 3 of sickness. After the initial fluid resuscitation in the ED, he developed a seizure followed by cardiopulmonary arrest on arrival to the ICU. The postmortem result of this patient showed evidence of encephalitis. Seven other cases (29.2 %) had evidence of hemodynamic instability as evidenced by poor perfusion, tachycardia or hypotension requiring initial fluid resuscitation but without evidence of myocarditis following further investigations. Two out of the 5 intubated patients had CXR changes suggestive of pulmonary edema following endotracheal intubation.

The presence of high fever and a longer duration prior to presentation were not significantly associated with a severe course (Table 1). Neither did the presence of positive contact history (56.5 % versus 41.7 %, *p* = 0.236) nor vomiting (41.7 % vs 33.3 %, *p* = 0.494) predict for severe disease. The severe cases were, however, associated with symptoms of hypoperfusion (46 % vs 17 %, *p* = 0.012), seizure (50 % vs 2 %, *p* < 0.001) and altered mentation (33 % vs 2 %, *p* = 0.016) in the presenting history.

**Table 1:** Demographic data and presenting history

	Cases (*n* = 24)	Controls (*n* = 48)	OR (95 % CI)	*P* value^a^
Demographics				
Age (year) median (IRQ)	3.0 (1.5;5.5)	2.5 (1;5.5)	1.04 (0.70;1.55)	0.848
Female	15 (63 %)	26 (54 %)	0.72 (0.27;1.92)	0.506
Presenting History				
Level of fever (°C) (*N* = 23 vs 42) median (IQR)	39 (38.7;39.4)	39 (38.5;39.5)	1.06 (0.49;2.25)	0.888
Temperature ≥ 39 °C	17 (74 %)	24 (57 %)	2.19 (0.69;6.96)	0.183
Temperature < 39 °C	6 (26 %)	18 (43 %)		
Duration of fever (days)	3.3 (2.0)	2.4 (1.6)	1.27 (0.97;1.67)	0.077
Contact history (*N* = 23 vs 48)	13 (57 %)	20 (42 %)	1.94 (0.65;5.79)	0.236
Vomiting	10 (42 %)	16 (33 %)	1.42 (0.52;3.86)	0.494
Symptoms of hypoperfusion^c^	11 (46 %)	8 (17 %)	NC	0.012^b^
Seizure	12 (50 %)	1 (2 %)	NC	<0.001^b^
Altered mentation	8 (33 %)	3 (6 %)	13.11 (1.61;106.60)	0.016

*NC* non-computable

^a^from conditional logistic regression

^b^from Fisher’s exact test

^c^as characterized by lethargy, syncope or reported cool extremities

Comparing the initial vital signs on arrival (Table 2), children with severe HFMD had a significantly higher heart rate and respiratory rate, especially among infants and children aged less than 5 years. However, there was no significant difference in the blood pressure at presentation. The severe cases had a higher incidence of having GCS of less than 15 on arrival (Table 3). Four had deterioration in GCS in the ED subsequently. All patients in the control group had normal GCS with no subsequent deterioration. Higher incidence of seizure, altered mental state, poor peripheral perfusion and meningeal irritation were also observed among the severe cases. More controls than cases presented with the full spectrum of typical rash on the hands and feet, and with oral ulcers. However, the absence of oral ulcers was not associated with severe disease (Table 3).

**Table 2:** Vital signs at presentation

Initial vital signs	Cases (*n* = 24)				Controls (*n* = 48)					
	Median (IQR)				Median (IQR)				OR (95 % CI)	*P* value
	<1 year	1- 4.99 years	5-9.99 years	>10 years	<1 year	1- 4.99 years	5-9.99 years	>10 years		
	*n* = 3	*n* = 14	*n* = 5	*n* = 2	*n* = 6	*n* = 28	*n* = 10	*n* = 4		
Heart rate (beats/min)	184 (170;200)	152 (131;173)	115(104;119)	117 (81;153)	146 (120;178)	123 (109;157)	112 (109;120)	109.5 (86.5;116.0)	1.02 (1.00;1.05)	0.021
Respiratory rate (breath/min)	40 (36;46)	30 (26;32)	25 (22;28)	31 (18;44)	35 (34;38)	30 (24;30)	20 (20;22)	20 (18;21)	1.15 (1.01;1.29)	0.03
Systolic blood pressure (mmHg) (*N* = 24 vs 46)^a^	100 (75;111)	106 (96;118)	114 (91;121)	120.5 (108;133)	106 (87;110)	100 (87;108)	107.5 (100.0;129.0)	114 .0(104.5;115.5)	1.03 (0.99;1.07)	0.16
Diastolic blood pressure (mmHg) (*N* = 24 vs 46)^a^	50 (49;67)	64.5(53.0;78.0)	65 (60;71)	65 (62;68)	54 (46;63)	65 (58;70)	66 (57;68)	68.5 (63.5;73)	1.01 (0.96;1.05)	0.819
Pulse oximetry reading (%)	97 (96;100)	98.5 (98.0;100.0)	100 (100;100)	96.5 (95;98.5)	100 (99;100)	99 (98;100)	99 (99;99)	100 (99;100)	0.69 (0.48;0.97)	0.032
Temperature on arrival (°C)	38.40 (37.90;39.50)	38.55 (37.40;39.10)	36.3 (36.3;37.6)	36.8 (36.5;37.1)	38.05 (37.4;38.0)	37.20 (36.85;37.95)	37.85 (37.1;38.9)	37 (36.7;37.1)	1.32 (0.84;2.09)	0.227

^a^No documented blood pressure for 2 controls

**Table 3:** Clinical signs at presentation

	Cases (*n* = 24)	Controls (*n* = 48)	*p* value^a^
GCS < 15 at presentation	7 (29 %)	0 (0 %)	<0.001
Deterioration in GCS^a^	4 (17 %)	0 (0 %)	0.010
Seizure on arrival	6 (25 %)	0 (0 %)	0.001
Altered mentation	11 (46 %)	0 (0 %)	<0.001
Poor peripheral perfusion	8 (33 %)	0 (0 %)	<0.001
Distribution of rash and presence of ulcers (*N* = 23 vs 48)			
Typical hands and feet rash and oral ulcers	15 (63 %)	41 (85 %)	0.037
Absence of oral ulcers	6 (25 %)	4 (8 %)	0.066
Meningeal irritation	4 (17 %)	0 (0 %)	0.010

^a^Any drop in GCS

Raised total white blood cell count (14.54 vs 8.27, *p* = 0.003) and absolute neutrophil count (9.08 vs 4.49 *p* = 0.004) were associated with a more severe course of HFMD (Table 4). There was a higher rate of EV-A71 positivity among cases than controls (38 % vs 15 %, *p* = 0.031). Most of the positive swabs were obtained from the throat. Hyperglycaemia (glucose >8.3 mmol/L, or 150 mg/dL) were observed in 3 out of 17 severe cases with documented blood glucose. Among the cases, 15 (62.5 %) had a lumbar puncture performed. There was no bacterial growth from the cerebrospinal fluid samples obtained. Six patients (25.0 %) had a magnetic resonance imaging performed, of which 3 revealed abnormal signals in the posterior or temporal parietal regions.

**Table 4:** Results of investigations

Investigations	Cases (*n* = 24)	Controls (*n* = 48)	OR (95 % CI)	*P* value
Initial Full Blood Count (*N* = 24 versus 31)				
Total white cell count, median (IQR)	14.54 (11.0; 18.20)	8.27 (7.02;8.27)	1.36 (1.11;1.66)	0.003
Absolute neutrophil count, median (IQR)	9.08 (6.51;12.09)	4.49 (3.37;4.49)	1.38 (1.11;1.71)	0.004
Absolute lymphocyte count, median (IQR)	2.81(2.39;4.43)	2.60(2.07;2.60)	1.28 (0.89;1.85)	0.18
EV-A71 Status				
Positive	9/24 (38 %)	7/48 (15 %)	10.28 (1.24;85.21)	0.031
Stool N (%)^a^	2/9 (22 %)	1/7 (14 %)		
Throat swab N (%)^a^	8/9 (89 %)	7/7 (100 %)		
CSF N (%)^a^	0/9 (0 %)	0/9 (0 %)		

^a^Numbers are accounted for because some patients had more than 1 specimen site tested

## Discussion

In this study, we identified 8 risk factors associated with severe HFMD, namely the presence of evidence of hypoperfusion, seizure, altered mentation (both on the presenting history and physical examination), meningeal irritation, tachycardia, tachypnea, raised total white blood cell (specifically absolute neutrophil count) and EV-A71 positivity. Evidence of hypoperfusion, increased respiratory rate, raised total white blood cell and positive EV-A71 are consistent with other studies [7–9, 11]. Out of these risk factors, seizure, altered mentation and meningeal irritation, are themselves signs of CNS complications.

More than a decade ago in a study done in our population [5], atypical physical findings, raised total white cell count, vomiting and the absence of mouth ulcers were reported as predictive factors for a fatal course of HFMD. This study, which involved a total of 138 patients with 7 fatalities, compared the fatal and non-fatal cases of HFMD. In our current study comparing a larger number of severe HFMD patients to controls, vomiting and absence of oral ulcers, did not reach statistical significance. We found only one fatality related to severe HFMD over the last one decade. This is significantly lower than the seven fatalities reported by Chong et al. [5] during and after the epidemic of HFMD in 2000. Since then, the mortality and overall morbidity has significantly reduced in our country. This is very likely to be contributed by the better infectious disease control measures instituted especially at the level of childcare centres, greater awareness and earlier health seeking behavior.

We observed that the majority (75 %) of the severe HFMD were children less than 5 years old. Young age has been reported to be associated with severe HFMD [8, 11, 13, 14]. This could be attributed to the low herd immunity in children less than 5 years old [15].

Recent studies surveying risk factors for severe HFMD have emerged [7–9] mainly from China, which has a high disease burden from HFMD [1]. A recent meta-analysis involving 19 separate studies [11] found that clinical characteristics such as young age, home care, duration of fever ≥ 3 days, body temperature ≥ 37.5 C, lethargy, vomiting, hyperglycemia, increased neutrophil count and EV-A71 infection were significantly related to the risk of severe HFMD. Our population differs from the HFMD population in China, in that our prevalence of severe cases tends to be lower, our patients are older [7, 8], and we have a lower incidence of EV-A71 infection [9].

One study in China involving 176 children, aged 6 to 45 months, demonstrated a relatively high incidence of severe HFMD complicated by cardiopulmonary collapse (18 %). This study observed that children with severe HFMD and circulatory collapse often had high blood pressure and heart rate in the early stage. The two patients with CNS involvement in our study who had cardiopulmonary collapse demonstrated tachycardia with normal blood pressure on arrival at the ED. Tachycardia can be a sign of autonomic dysfunction from CNS complications or may be a sign of cardiac involvement. Screening children with severe HFMD for these abnormal vital signs is important in predicting impending cardiorespiratory failure and allowing the timely initiation of appropriate interventions. Such patients should receive close inpatient monitoring by nursing staff who are familiar with age-appropriate vital sign norms, frequent charting of vital signs and GCS, and an early consideration of the need for hemodynamic support.

The 2 patients who had evidence of pulmonary edema on CXR had encephalitis with cardiopulmonary collapse. Both of them received a total of 40 ml/kg of resuscitation fluids each during the initial stage. Damage to certain areas in the brain stem in encephalitis can cause neurogenic pulmonary edema. Several mechanisms that have been proposed to explain the pathogenesis of neurogenic pulmonary edema include an increase in pulmonary vascular pressure and an increase in pulmonary endothelial permeability [16]. Fulminant neurogenic pulmonary edema had been reported in patients who died from HFMD in Malaysia and Taiwan [13, 17]. In view of the risk of neurogenic pulmonary edema in HFMD patients with CNS involvement, fluid resuscitation, when indicated, should be given judiciously to prevent fluid-overloading of the lungs.

We acknowledge the limitations of our study. Firstly, a case–control methodology was chosen because of the low event rate of severe HFMD in our population. Across the years, there may have been differences in the diagnosis and management of these patients. Also, by age-matching, we sought to search for red flags in the respective age strata that would guide the ED physician. By age-matching, however, the effect of age on the respective variables could not be studied. With retrospective studies, results could be confounded by information bias (chart reviewers were not blinded to the objective of the study). Finally, given the small number of cases, it was statistically inappropriate to perform a multivariable analysis to derive independent risk factors.

## Conclusions

The presence of evidence of hypoperfusion, tachycardia, and tachypnea may herald severe disease and should alert physicians assessing young children with HFMD. Physicians should be particularly cognizant of signs of CNS complications among children with HFMD, especially seizure, altered mentation and meningeal irritation. Particular care should be taken in the presence of raised total white blood cell (specifically absolute neutrophil count) and EV-A71 positivity. Patients with such risk factors should be observed closely with careful monitoring of their vital signs and GCS. Any clinical deterioration should prompt early institution of supportive care eg airway support, measured fluid resuscitation and inotropic support. In addition, we propose that in patients with likely central nervous system involvement, fluids should be managed judiciously because of their risk for pulmonary edema. Future work in this area should include comparative studies of severe HFMD disease in different populations.

## Abbreviations

HFMD: Hand foot and mouth disease
EV-A71: Enterovirus A71
ICD: International Classification of Diseases
AFP: Acute flaccid paralysis
CXR: Chest X-ray
GCS: Glasgow coma scale
IQR: Inter-quartile range
ICU: Intensive care unit
ED: Emergency department
CNS: Central nervous system
